# A novel approach to identifying regulatory motifs in distantly related genomes

**DOI:** 10.1186/gb-2005-6-13-r113

**Published:** 2005-12-30

**Authors:** Ruth Van Hellemont, Pieter Monsieurs, Gert Thijs, Bart De Moor, Yves Van de Peer, Kathleen Marchal

**Affiliations:** 1 ESAT-SCD, KU Leuven, Kasteelpark Arenberg 10, 3001 Leuven-Heverlee, Belgium; 2 Plant Systems Biology, Bioinformatics and Evolutionary Genomics, VIB/Ghent University, Technologiepark 927, 9052 Gent, Belgium; 3 Department of Microbial and Molecular Systems, KU Leuven, Kasteelpark Arenberg 20, 3001 Leuven-Heverlee, Belgium

## Abstract

Although proven successful in the identification of regulatory motifs, phylogenetic footprinting methods still show some shortcomings. To assess these difficulties, most apparent when applying phylogenetic footprinting to distantly related organisms, we developed a two-step procedure that combines the advantages of sequence alignment and motif detection approaches. The results on well-studied benchmark datasets indicate that the presented method outperforms other methods when the sequences become either too long or too heterogeneous in size.

## Abstract 2

A two-step procedure for identifying regulatory motifs in distantly related organisms is described that combines the advantages of sequence alignment and motif detection approaches.

## Background

Phylogenetic footprinting is a comparative method that uses cross-species sequence conservation to identify new regulatory motifs [1]. Based on the observation that functional regulatory motifs evolve more slowly than non-functional sequences, the method identifies potential regulatory motifs by detecting conserved regions in orthologous intergenic sequences [2,3]. The comparison of orthologous sequences from multiple genomes is often based on multiple sequence alignment [4,5] and several alignment algorithms, such as CLUSTALW [6], DIALIGN [7,8], MAVID [9,10] and MLAGAN [11], have proven very useful to identify conserved motifs in closely related higher vertebrate sequences [4,12,13]. Although the comparison of closely related organisms has proven successful, inclusion of more distantly related species can greatly improve the detection of conserved regulatory motifs. By adding more distantly related sequences, the conserved functional motifs can be more easily distinguished from the often highly variable 'background' sequence. Moreover, this leads to the detection of motifs that have a function in a wider variety of organisms, for example, all vertebrates [14-19]. Both Sandelin *et al. *[20] and Woolfe *et al. *[21], for instance, performed a whole genome comparison of human and pufferfish, which diverged approximately 450 million years ago (mya) to discover non-coding elements conserved in both organisms. They showed that most of these conserved non-coding elements are located in regions of low gene density (implying long intergenic regions) [21]. Moreover, many of the conserved non-coding elements are located at large distances from the nearest gene [20,21]. These findings led to the conclusion that it is interesting to analyze whole intergenic regions of vertebrate genes, rather than limit the comparative analyses to the promoter region located near the transcription start.

However, vertebrate intergenic regions may differ considerably in size, such as when comparing intergenics of, for example, mammals with those of *Fugu *[22-24]. Since multiple sequence alignments are often based on global alignment procedures, they will likely fail to correctly align such sequences of heterogeneous length [25].

An alternative for alignment methods is the use of *de novo *motif detection procedures for phylogenetic footprinting. These are based on either probabilistic or combinatorial algorithms. One such method, FootPrinter [26,27], uses a string based motif representation with dynamic programming to search a phylogenetic tree for motifs that show a minimal number of mismatches. Probabilistic algorithms, such as MEME [28], Consensus [29,30] and Gibbs sampling [31,32], use a matrix representation of the motif (position specific weight matrix). Currently, several implementations of Gibbs sampling are available, such as AlignACE [33,34], ANN-spec [35], BioProspector [36] and MotifSampler [37-40]. However, these algorithms are sensitive to low signal-to-noise ratios, that is, the presence of small motifs (five to eight base pairs (bp)) in long intergenic sequences. This often results in the detection of many false positive motifs. On the other hand, an advantage of these procedures is that, because motif detection comes down to locally aligning the orthologous sequences, non-collinear motifs can still be detected.

Neither motif detection nor multiple alignment methods are optimally suited to correctly align long intergenic sequences of heterogeneous length. Here, we present a simple two-step procedure that identifies conserved regions by combining the advantages of both alignment and motif detection methods. Such highly conserved regions most likely contain transcription factor binding sites or other functional intergenic sequences [41]. To show its efficiency, we applied our two-step approach to well described benchmark datasets. Since regions of strong conservation among divergent vertebrates are often associated with developmental regulators [20,21], we choose mainly these types of genes to test our methodology. The presented approach, however, is applicable to any set of organisms and genes for which one wants to compare the intergenic sequences.

## Results

### A two-step procedure for phylogenetic footprinting

In this study, we aimed to detect regulatory motifs that have been retained over long periods in evolution; in our test case, this applied to mammals to ray-finned fishes such as *Fugu*. The *Fugu *genome, however, is very compact and approximately eight or nine times smaller than the human one, although both genomes are assumed to contain a similar repertoire of genes. The compactness of the genome of *Fugu *is the result of shorter intergenic regions and introns [22,23,42]. On the other hand, the preliminary and still often erroneous annotation of the *Fugu *genome sometimes results in the selection of very long intergenic regions. Such heterogeneous sizes of the intergenic regions that need to be compared complicate identification of regulatory motifs. Widely used alignment algorithms, such as AVID, LAGAN and others, will usually fail when the sequences that need to be aligned differ too drastically in length. This problem is exacerbated when the sequences have a low overall percent identity. To cope with this, motif detection procedures could offer a solution. However, because regulatory motifs are typically only 6 to 30 bp long, whereas intergenic sequences of vertebrate genes range up to tens of kilobases [43], this results in a low signal-to-noise ratio that complicates the immediate use of *de novo *motif detection procedures. Therefore, we developed a two-step procedure to combine the advantages of the alignment and motif detection procedures.

**Figure 1:**
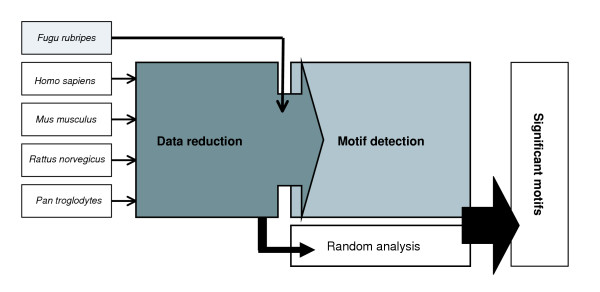
Schematic representation of the two-step procedure for phylogenetic footprinting. In the data reduction step, regions conserved among closely related (mammalian) orthologs are selected. Subsequently, these strongly conserved sequences are combined with a more distant ortholog (for example, *Fugu*); this set of genes is then subjected to motif detection. Finally, significantly conserved blocks are identified using a threshold defined by a random analysis.

We included a first data reduction step based on an alignment method prior to the second motif detection step (see Materials and methods and Figure 1). This data reduction step increases the signal-to-noise ratio in the input set used for motif detection. Data reduction is based on the assumption that longer regions conserved in the orthologs of closely related species are more likely to contain biologically relevant motifs compared to non-conserved regions [21]. Therefore, in our benchmark study, regions conserved among closely related orthologous intergenic sequences of comparable size were preselected as input for motif detection. The mammalian intergenic sequences showed a relatively high overall percent identity and were comparable in length. Subsequently, these selected conserved mammalian subsequences were subjected to motif detection, together with the full-length *Fugu *intergenic region.

### Data reduction

The data reduction procedure preselects subsequences conserved in closely related (mammalian) sequences. It requires a multiple alignment procedure that combines a pairwise alignment (AVID) and a clustering algorithm (Tribe-MCL). Details on this procedure can be found in the Materials and methods section. A resulting cluster consists of unique, non-overlapping subsequences, corresponding to a specific region conserved among the different related orthologs (human, chimp, mouse and rat).

In our benchmark study, we were primarily interested in finding DNA motifs conserved among all input sequences (orthologs). Therefore, only clusters containing conserved subsequences of all mammalian orthologs included in this study (human, chimp, rat and mouse) were retained for further analysis (supplementary website [44]).

### Motif detection

The motif detection step aims at identifying motifs that are statistically over-represented in the reduced set of orthologous intergenic sequences. To this end, we extended a previously developed Gibbs sampling based motif detection approach, MotifSampler [37-39] (see Materials and methods). The adapted implementation allows the user to choose a core sequence. A potential motif is only retained when it occurs in this core sequence. Indeed, the input data for motif detection consists of a set of (mammalian) subsequences and a complete *Fugu *intergenic sequence. This *Fugu *sequence shows a relatively low overall percent of identity with the other sequences. Due to the high sequence conservation (strong data dependence) between the mammalian subsequences, the original implementation of MotifSampler is not appropriate for detecting motifs in the most divergent sequence: the cost function (log likelihood score) that is optimized in the original MotifSampler offers a trade-off between the degree of conservation of the motif and the number of occurrences of the motif [45]. This results in the detection of motifs that are highly conserved between the highly similar (mammalian) sequences but that show little or no conservation with the *Fugu *intergenic sequence. Therefore, to ensure the detection of motifs conserved among all sequences, we introduced the concept of a core sequence. By selecting the most divergent ortholog (the *Fugu *sequence) as the core sequence, the algorithm is forced to only detect motifs that are also present in the most distantly related organism.

The adapted implementation was also redesigned to search for long conserved blocks instead of searching for short conserved motifs only. In datasets consisting of orthologs, not only the motif itself is conserved but also the local context of the motif [21,45]. For this reason, we designed BlockSampler to extend motifs and search for the longest conserved blocks. A motif is thus used as a seed to generate ungapped multiple local alignments. Looking for longer motifs/blocks also increases the specificity of motif detection (less false positives). Finally, since it was previously shown that choosing a background model increases the performance of motif detection [37], we adapted the algorithm such that it uses for each ortholog in the dataset an organism-specific background model.

### Results of developed methodology on benchmark datasets

To evaluate its performance, we applied our two-step motif detection procedure to several benchmark datasets. Since we were primarily interested in detecting regulatory motifs over large evolutionary distances, that is, conserved between *Fugu *and mammalian genomes, we compiled sets of evolutionarily divergent vertebrate orthologs that had been described to contain conserved motifs.

In vertebrate organisms, large conserved regions tend to be associated with genes encoding regulators of development [20,21]. Since our strategy aims at detecting such conserved blocks, we tested the methodology on three sets of orthologous genes that function in the regulation of development, containing motifs described in the literature: *hoxb2 *[46], *pax6 *[47] and *scl *[48]. We also included in the analysis one gene, *cfos*, not related to developmental processes [26].

All the benchmark sets consisted of orthologous genes that contain evolutionarily retained motifs described in the literature that have, to a large extent, been experimentally verified. These known motifs were used to evaluate the performance of our approach and to compare it to other algorithms. Additionally, we monitored whether our procedure was capable of detecting as yet unknown motifs.

**Figure 2:**
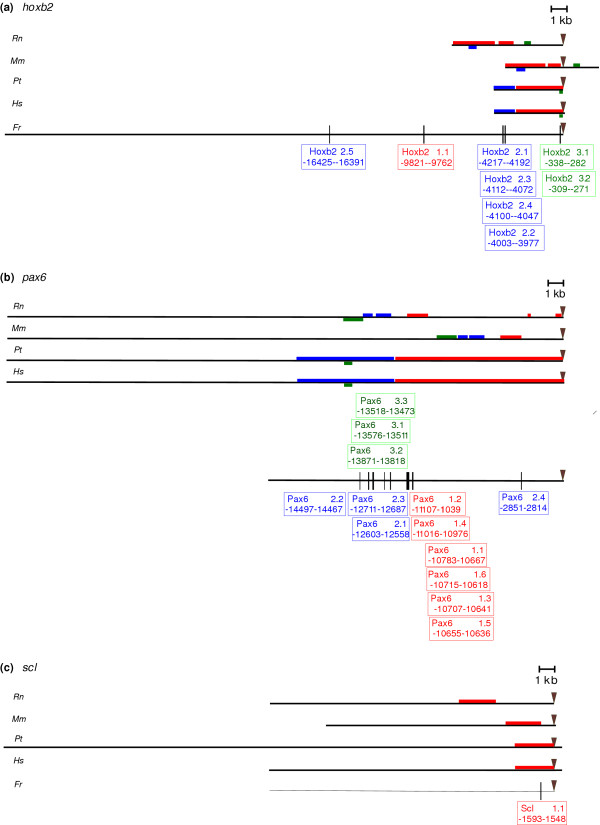
Localization of clusters and conserved blocks in the **(a) ***hoxb2*, **(b)***pax6 *and **(c)***scl *datasets. For each dataset, the different orthologous intergenic sequences are shown: *Rn*,*Rattus norvegicus*; *Mm*, *Mus musculus*; *Pt*, *Pan troglotydes*; *Hs*, *Homo sapiens*; *Fr*, *Fugu rubripes*. Clusters of conserved mammalian subsequences that were subjected to motif detection (that is, clusters containing at least one subsequence per mammalian organism) are represented on the respective mammalian sequences (cluster 1 in red, cluster 2 in blue and cluster 3 in green). The conserved blocks identified using BlockSampler are represented on the *Fugu *intergenic sequence (in the color of the mammalian cluster it is located in). For each block the localization relative to the start of the *Fugu *gene is given. The transcription start sites are marked with an inverse triangle.

Using the two-step procedure we detected 8 significant blocks for *hoxb2*, 13 for *pax6*, 1 for *scl *and none for the *cfos *dataset (Table 1). The consensus scores of each of these 22 blocks are given in Tables 2, 3, 4 for each benchmark dataset, respectively. The location of these blocks on the complete intergenic region of the respective *Fugu *orthologs is shown in Figure 2; alignments can be found in [44].

As a first validation step, we compared our results with the alignments and conserved regions identified by well-established genome browsers, namely the UCSC genome browser [49] and the UCR browser [20] (Table 1).

The UCSC genome browser [50] enables access to current genome assemblies; it offers visualizations of several genomic features, such as cross-species homologies [49,51]. The latter can be viewed as multiple alignments over several species, ranging from closely related mammals to more distantly related species, such as chicken, zebrafish and pufferfish. The multiple alignments were generated with MULTIZ [52]. Of the conserved 22 blocks we identified by aligning intergenic regions of mammals and *Fugu*, 16 could also be retrieved from the USCS genome browser (Table 1); these are indicated in Tables 2, 3, 4. The remaining six blocks could only be identified using our two-step approach.

The set up of the UCR browser [53] is slightly different from the UCSC browser in that it focuses on the detection of ultra-conserved regions (UCRs) only, that is, regions conserved between human, mouse and *Fugu*. These regions were identified using sequence alignment strategies (BLAT) applied to complete genome sequences without prior data reduction [20,54]. Although our strategy also identifies regions highly conserved among the species under study, no overlap was detected between our conserved blocks and the UCRs (Table 1); that is, in the regions we studied (up to 40 kb intergenic plus 5' untranslated region), no UCRs were located according to the analysis of Sandelin *et al. *[20]. The regions the UCR browser identified as ultra-conserved were located much more upstream of the gene compared to the regions we used for our analysis.

To further validate the detected blocks, we tested whether they contain the motifs that were originally reported by Scemama *et al. *[46], Kammandel *et al. *[47] and Göttgens *et al. *[48] for *hoxb2*, *pax6 *and *scl*, respectively (no significant blocks were detected for *cfos*). The previously described motifs present in the respective blocks are listed in Tables 2, 3, 4 (marked with an asterisk). Of the 17 motifs reported by Scemama *et al. *[46], 8 were present in the significant *hoxb2*-blocks (Table 2). Five other motifs were present in non-significant blocks. The latter are blocks with scores that fell below the threshold we chose based on the random analysis (see Materials and methods). The four remaining motifs could not be recovered. All motifs described by Kammandel *et al. *[47] as conserved among mammalian and *Fugu pax6 *intergenic regions were recovered by our methodology (Table 3). The conserved block detected in the *scl *dataset contains three of the five motifs previously identified by Göttgens *et al. *[48] (Table 4); a fourth motif was picked up in a non-significant block. One motif was not detected in any of the blocks.

Besides these blocks containing known motifs, we identified several blocks (three for *hoxb2 *and eight for *pax6*) that correspond to conserved regions not previously described in the literature. To validate these blocks, we checked whether they were enriched for yet undescribed regulatory motifs. Hence, we screened all blocks with the Transfac database of vertebrate transcription factor binding sites [55]. The result of this screening is summarized in Tables 2, 3, 4. As expected [41,56], the conserved blocks we identified contain many potential binding sites; remarkably they tend to be specifically enriched for homeodomain binding sites (in blocks hoxb2 1.1, hoxb2 2.1, hoxb2 2.3, hoxb2 2.4, pax6 1.1, pax6 1.4, pax6 3.1, pax6 3.3 and scl 1.1, homeodomain binding sites were significantly over-represented, with a *p* value < 10^-8^). For a more detailed description of both the previously described and the new potential regulatory motifs present in the detected blocks, please refer to the Supplementary website [44].

Besides these well-described benchmark datasets, we applied our method to six additional datasets, differing in composition from the benchmark datasets. They all contained a combination of four mammalian sequences (rat, mouse, human, chimp or dog) to be used in the data reduction step and an additional set of sequences originating from more distantly related orthologs (chicken, *Fugu*, *Tetraodon nigroviridis *and zebrafish in different combinations) added in the motif detection step. Four of the six additional datasets were derived from genes functioning in developmental regulation, including three homeobox genes (*GSH1*, *Meis2*, *HOXB5*) and one encoding the zinc finger protein EGR3. Besides these regulators involved in development, two genes, *PCDH8 *and *HIV-EP1*, were included, which are, according to our knowledge, unrelated to development. PCDH8 is believed to function as a calcium-dependent cell-adhesion protein and HIV-EP1 binds to enhancer elements present in several viral promoters and in a number of cellular promoters such as those of the class I MHC, interleukin-2 receptor, and interferon-beta genes. In the additional datasets involved in development, we detected several strongly conserved blocks: *GSH1 *contained four blocks that are conserved among human, chimp, mouse, rat and pufferfish (*Fugu *and *Tetraodon*); in *Meis2*, two blocks were recovered that are retained in all organisms under study except for *Fugu*; and in *HOXB5*, six strongly conserved blocks were detected in mammals and pufferfish, while the motif seems to have been lost in chicken. In *EGR3*, two blocks were found conserved in mammals and fish. In the non-developmental related datasets, only in *PCDH8 *was one large block detected, conserved in human, chimp, mouse, rat, chicken, *Tetraodon *and *Fugu*, but not in zebrafish. This shows that conserved regions might also exist in genes not involved in development, although a possible involvement of this additional gene in developmental processes cannot be ruled out. Detailed results of these analyses can be found in Additional data file 1 and in [44]. Because the motifs in these additional datasets have not been studied as extensively as those of the benchmark datasets, we cannot guarantee all detected blocks are biologically functional.

### Evaluation of the developed procedure

To compare the performance of our newly developed two-step strategy to that of other frequently used algorithms, we evaluated to what extent MotifSampler [39], MAVID [10] and 'Threaded Blockset Aligner' (TBA) [52] could recover known motifs in our benchmark sets.

First, we studied the performance of the alignment algorithms MAVID and TBA in detecting conserved regions within our four benchmark datasets. Since MAVID and TBA were originally developed to perform multiple alignments on long sequences, we applied these algorithms to the initial full-length benchmark datasets, that is, the complete mammalian and *Fugu *intergenics. We evaluated to what extent motifs or conserved regions described in original articles were correctly aligned using either MAVID or TBA. The results are summarized in Table 5 (MAVID and TBA columns) and in [44].

**Figure 3:**
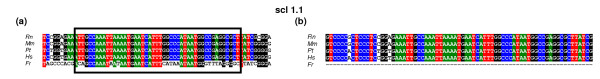
Comparison of two-step strategy with MAVID for the *scl *data set **(a) **Conserved block: alignment of the different *scl *orthologs. The conserved block as identified by BlockSampler - is marked with a boxed area. **(b) **Visualization of the MAVID alignment of the corresponding region. The dashed line denotes a gap in the alignment. *Rn*, *Rattus norvegicus*; *Mm*, *Mus musculus*; *Pt*, *Pan troglotydes*; *Hs*, *Homo sapiens*; *Fr*, *Fugu rubripes*.

MAVID alignment of all three *cfos *datasets (mammalian orthologs combined with each of the three *Fugu *paralogs) could not recover either of the two motifs previously described by Blanchette and Tompa [26] (Table 5). This is in line with our results showing the overall low homology between the *cfos *mammalian and *Fugu *orthologs. The MAVID alignment of most of the *hoxb2 *blocks containing previously described motifs shows that a conserved region in the mammalian intergenic sequences is broken up into small conserved parts interrupted by gaps when aligned to the longer *Fugu *sequence, resulting in an incorrect alignment of the regulatory motifs: previously reported motifs were not recovered in the MAVID alignment (Table 5). Our method performs better because the most heterogeneous sequence is only aligned in a second step, using a highly flexible local alignment procedure (BlockSampler). Regarding *pax6*, most of the blocks containing previously described motifs were correctly aligned by MAVID and all the motifs described by Kammandel *et al. *[47] could be correctly retrieved over all the orthologs under study (Table 5). This dataset is probably relatively well suited for MAVID because the mammalian sequences are only twice as large as the pufferfish *pax6 *intergenic region (Table 6). Although the lengths of the intergenic regions in the *scl *dataset (Table 6) are in the same order of magnitude (ranging from 16.5 to 40 kb), MAVID did not succeed in identifying any of the motifs previously described by Göttgens *et al. *[48] (Figure 3, Table 5).

Although TBA has been shown to outperform MAVID in aligning more divergent sequences [52], applying this alignment tool to the benchmark datasets generated similar results as MAVID: all known *pax6*-regulating motifs were detected, while motifs present in the other benchmark datasets were not recovered (Table 5, TBA column).

Besides detecting the blocks with previously described motifs, our two-step methodology also discovered blocks (block pax6 2.4, for instance) that could not be recovered when aligning the intergenic sequences with MAVID or TBA [44,57].

Overall, based on our benchmark analysis, the two-step method performs better than MAVID or TBA in identifying conserved blocks in distantly related orthologs: the proposed method is able to recover in our benchmark sets all the known motifs identified by MAVID and TBA but, in addition, finds several previously described motifs ignored by these algorithms (Table 5, two-step BS, MAVID and TBA columns). Using the two-step procedure, first selecting strongly conserved orthologous sequences, clearly facilitates alignment with the more divergent (lower overall similarity) sequence.

We also tested the performance of MotifSampler as an example of a probabilistic motif detection procedure on the unreduced dataset. In this case, only one previously described motif was detected (Table 5, MS column). This was to be expected as in unreduced datasets the signal to noise ratio is too high for standard motif detection procedures to give reliable and interpretable results.

**Figure 4:**
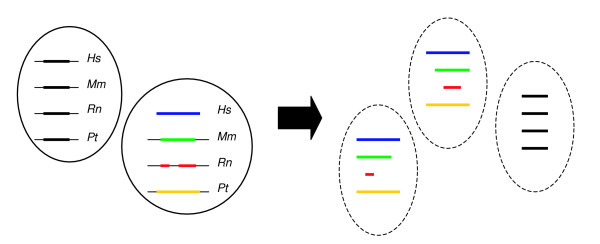
Schematic representation of subclusters, that is, clusters of conserved orthologous sequences that contain one region in each ortholog. See text for details. *Rn*, *Rattus norvegicus*; *Mm*, *Mus musculus*; *Pt*, *Pan troglotydes*; *Hs*, *Homo sapiens*.

Our two-step procedure includes two adaptations over previous existing methods: first, it allows for a data reduction step; and secondly, we developed a motif detection procedure specifically adapted to the purpose of detecting large conserved blocks (BlockSampler). To assess the relative contribution of each of these adaptations to the overall result, we set up the following experiment: to study the specific influence of the data reduction step, we compared the results of applying BlockSampler to both the unreduced benchmark datasets and the datasets obtained after data reduction. Table 5 (BS and two-step BS columns) shows the results of this comparison. Overall, the results seem comparable: application of BlockSampler to the complete intergenic sequences results in recovery of 15 of the 30 previously reported motifs (in all four datasets), while the two-step method identified 17. Thus, at first sight, there does not seem to be a major contribution from the data reduction step. A closer look at Table 5, however, shows that the positive contribution of the data reduction (increasing the signal-to-noise ratio) is strongly dependent on the lengths of the intergenic sequences to be aligned. A major positive effect is observed for the large *pax6 *and *scl *datasets, whereas for the *hoxb2 *set, in which the sequences under study are rather short, the data reduction does not offer a clear advantage. To assess the specific improvements of using BlockSampler instead of standard motif detection approaches, we compared the results of BlockSampler to those of MotifSampler when both were applied to the reduced datasets. A reduced dataset thus consists of a subcluster of mammalian sequences (Figure 4) and a complete *Fugu *ortholog. The performance of MotifSampler was far below that of BlockSampler: MotifSampler only detected two previously described motifs (Table 5, two-step MS column), both in the *hoxb2 *set, while BlockSampler recovered 17 previously described motifs (Table 5, two-step BS column). Moreover, because MotifSampler searches for short motifs (default eight nucleotides (nt)), it detects many false positive hits. These results show that independent of the data reduction step, BlockSampler is clearly more suited for detecting large conserved blocks than MotifSampler.

## Discussion

We developed a two-step methodology to search for regions (motifs) conserved over different phylogenetic lineages in long intergenic sequences of heterogeneous size. In a first step, an alignment method is used to select conserved subsequences in intergenic orthologous sequences of comparable size of closely related vertebrate genomes, since these are expected to be enriched for regulatory motifs [21,41]. The combination of this preselected dataset of conserved sequences and the full-length intergenic sequence of a more distant ortholog, which is more likely to differ in size and overall homology, is subjected to probabilistic motif detection. The preselection step facilitates motif detection by enhancing the signal-to-noise ratio in the dataset. For the second motif detection step we used an extension of a Gibbs sampling based algorithm [39] with a higher performance in detecting large conserved blocks within a set of orthologous sequences. Using the strategy mentioned above, we could combine the advantages of alignment methods, which have been shown to be very suitable for aligning long, highly conserved intergenic sequences, and the probabilistic algorithms for motif detection that usually are more appropriate when looking for smaller regions of conservation (lower degree of similarity).

We applied this two-step methodology to four well-studied datasets for which functional phylogenetically conserved motifs had been extensively described. Our approach identified most of the previously described motifs. In addition, we detected several blocks not previously described in the literature or not present in any of the two genome browsers (UCSC and UCR) we compared our results with. Because highly conserved blocks most probably consist of consecutive transcription factor binding sites [21,41,56], we screened the conserved blocks with the Transfac motif database [55]. These blocks contained abundant copies of homeodomain binding sites. This is not unexpected since most of the genes we were studying function in the regulation of development [21,58]. These blocks most probably contain, besides the motifs obtained with the Transfac screening, many more motifs not yet annotated in Transfac. Alternatively, they might have other, not yet characterized biological functions, for example, transcripts of unknown function [59].

Some previously described motifs were missed, however, because of the strong selection criteria we used: since regulatory elements tend to be grouped [21,41,56,60], we assumed that the sequences surrounding a regulatory motif are also conserved (due to the presence of other binding sites). Motifs located in a variable context will probably go undetected.

By applying our method to additional datasets with configurations different from the benchmark dataset we could demonstrate that our methodology is more generally applicable.

Comparing the performance of the two-step procedure with that of MAVID and TBA, as representatives of multiple alignment methods, and MotifSampler, as an example of a motif detection method, showed that our approach outperformed these alternative methods when the intergenic sequences became either too long or too heterogeneous in size.

Additionally, we studied the marginal contribution of the data reduction step and the improved method for motif detection on the final performance of the two-step procedure: overall, BlockSampler performed better than the related algorithm MotifSampler, both on long sequences and on intergenic regions reduced in size. The data reduction step seemed essential when the length of the intergenic sequences to be compared becomes excessive.

Although our two-step procedure has proven successful, there is still room for improvement, for instance by taking into account the phylogenetic relationships between the sequences under study in the second motif detection step. The contribution of finding a motif in an ortholog to the global motif score could be weighted according to its phylogenetic distance from the other sequences in which the motif is also present. Indeed, this way we would account for the specific composition of a dataset because closely related orthologs are less informative than further related ones. If one wanted to relax the assumption of conserved order of motifs in the first data reduction step, it would suffice to replace AVID in this step with a more local aligner such as BLAT [54]. Also, our motif detection algorithm could be extended for more advanced background models [61].

## Conclusion

We developed a two-step approach that combines the advantages of both motif detection and multiple alignment algorithms. It has shown to be well suited for identifying conserved regions in intergenic sequences from distantly related orthologs that show a low overall homology and that are heterogeneous in size. The strength of our approach lies in the combination of data reduction and improved motif detection: the first data reduction step is essential when it concerns long intergenic sequences. BlockSampler, the algorithm used in the second motif detection step, has been shown to be optimally suited to identify large conserved regions among orthologous sequences. Applying our method to benchmark sets showed that, although it recovered most of the motifs/blocks previously described in these datasets, some were missed due to the assumptions underlying our analysis and the stringent selection criteria applied. These results indicate that, given the chosen criteria, our method offers a fully automated analysis flow that is highly specific for detecting motifs conserved over different vertebrate lineages in complete intergenic sequences.

## Materials and methods

### Benchmark datasets

The benchmark datasets were generated as follows. First, a set of orthologous genes was defined using the Ensembl genome browser version 23 [62]. In this study, the benchmark datasets included genes from human (*Homo sapiens*), mouse (*Mus musculus*), rat (*Rattus norvegicus*), chimp (*Pan troglodytes*) and pufferfish (*Fugu rubripes*). Regarding the *cfos *dataset, Ensembl identified three *Fugu *paralogs - SINFRUG00000132418, SINFRUG00000132419 and SINFRUG00000143787 - that were all included in the analysis. The additional datasets *EGR3, GSH1, HIV-EP1, HOXB5, Meis2, PCDH8 *contain multiple distantly related orthologs (see [44]).

Subsequently, the intergenic regions of these orthologs were selected using the Ensembl mart database release 21.1. The region upstream of the transcription start (as defined by Ensembl) was limited to 40 kb. Additionally, the 5' untranslated region was included. Lengths of the respective intergenics are given in Table 6; the benchmark datasets, *cfos*, *hoxb2*, *pax6 *and *scl *can be found in [44]. The rat *cfos *ortholog ENSRNOG00000008015, *Fugu hoxb2 *ortholog SINFRUG00000136637, chimp *pax6 *ortholog ENSPTRG00000003474, and *scl *chimp ENSPTRG00000003474 contain long N-stretches, probably as a result of incomplete preliminary annotation.

Remarkably, where *Fugu *is known to have a very compact genome [23], the *Fugu hoxb2 *mentioned above is very long compared to the mammalian *hoxb2 *intergenic sequences (Table 6). This is probably due to the presence of a pseudogene (SINFRUG00000157209) in the intergenic region of SINFRUG00000136637 at circa 5.9 kb from the transcription start site of *hoxb2*, which was not yet annotated in the release version 23 of ENSEMBL.

All intergenic sequences were selected as described above, except the intergenic sequence of the *Fugu scl *ortholog. Because the putative *scl *ortholog annotated by ENSEMBL (SINFRUG00000145588) did not contain motifs shown to be present in the *Fugu scl *ortholog by Göttgens *et al. *[48], we used the Genbank *Fugu scl *sequence [Genbank: AJ131019]. This sequence (referring to a cosmid sequence of circa 33 kb) was also used in the original study of Barton *et al. *[63]. To delineate the intergenic region of *scl*, we aligned the coding sequence from the *scl *homolog SINFRUG00000145588 with the AJ131019 sequence using 'blast 2 sequences' [64]. The coding region was located from positions 20,156 to 22,165; we then selected the upstream region (from positions 1 to 20,155).

### A two-step procedure for phylogenetic footprinting

A schematic representation of the developed two-step procedure is given in Figure 1.

#### Step 1: data reduction

In this step, a dataset consisting of the complete intergenic sequences of comparable size originating from orthologs of closely related organisms is reduced to a dataset of preselected sequences conserved among all/most compared orthologs. First, related vertebrate intergenic regions of comparable size (in this study these sequences corresponded to the mammalian human, chimp, rat, mouse and dog sequences) are aligned using the pairwise alignment algorithm AVID (using default parameters) [65]. For each ortholog, sequences corresponding to the significantly conserved regions of the pairwise alignment are selected using VISTA [66]. Significance of the alignment is defined by two parameters (VISTA parameters): the window length (L), the region for which the percent identity is calculated; and the conservation level (C) in the selected window, the minimal percent identity of the aligned region to be considered as significantly conserved. The parameter settings were adapted to the evolutionary distance of the compared organisms. The closer the organisms were related, the higher the threshold on the degree of conservation chosen. The conservation parameters used were: for human-mouse comparison, 85% over 200 nt; human-rat, 85% over 200 nt; human-chimp, 85% over 350 nt; human-dog, 80% over 200 nt; mouse-rat, 85% over 350 nt; mouse-chimp, 85% over 200 nt; mouse-dog, 80% over 200 nt; rat-chimp, 85% over 200 nt; rat-dog, 80% over 200 nt; and chimp-dog, 80% over 200 nt.

To identify orthologous regions conserved in multiple related vertebrate sequences of comparable size (that is, multiple alignment), homologies between all preselected sequences were determined (using AVID with default parameters). Subsequently, multiple conserved regions were identified using the graph based clustering TribeMCL [67]. We chose TribeMCL as this is a well-known graph-based clustering algorithm that was originally designed to recover transitivity relations between biological sequences (that is, orthologous proteins). Each resulting cluster corresponds to a region conserved in multiple sequences and consists of a set of preselected sequences originating from the different related orthologs of comparable size that mutually show a minimal degree of conservation. Several runs of TribeMCL were performed for each dataset, using different values of clustering parameters I and P (see [44]). The parameter I did not seem to have a major influence on the size of the clusters and, therefore, was set at 4. For the *P *value, three different values were tested per dataset and the parameter that resulted in small tightly linked clusters was chosen as these clusters correspond to strongly conserved regions. The parameters of choice for the benchmark datasets were: for *cfos*, I = 4 and *P *= 0; for *hoxb2*, I = 4 and *P *= -10; for *pax6*, I = 4 and *P *= 0; and for *scl*, I = 4 and *P *= -10. Concerning the additional datasets, the parameter setting of choice was I = 4 and *P *= 0 for *EGR3, HIV-EP1, HOXB5, Meis2 *and *PCHD8 *and I = 4 and *P *= -10 for *GSH1*.

Some clusters contain different subsequences derived from the intergenic sequence of a single organism that match one larger sequence of another organism; for example, two subsequences in rat that match one larger sequence in human. To minimize the noise in the datasets used for motif detection, such clusters are split into subclusters. Subclusters contain only a single subsequence of each ortholog (paralog; Figure 4). A subcluster is tagged by a profile containing the IDs of the different subsequences composing this subcluster. The input dataset for motif detection (Figure 1) thus consists of the mammalian subsequences in a subcluster together with the intergenic region of the corresponding *Fugu *ortholog.

#### Step 2: Motif detection

To find motifs conserved in the preselected intergenic sequences of orthologous genes, we developed BlockSampler as an extension of MotifSampler [39]. In contrast to the previous version of MotifSampler, which could only handle a single background model, in BlockSampler each orthologous intergenic sequence in the input dataset is scored with its appropriate species-specific background model. Previous studies have shown that using the correct species-specific higher order background model improves the reliability of the results [37,68]. In this study we used species-specific third-order background models.

The current implementation also allows selecting a user-defined core ortholog. This is the sequence of interest in which the motif should be present (in our case the sequence of heterogeneous length - the *Fugu *sequence). The idea behind this is that we are interested in motifs present in this core sequence that are supported by their presence in the preselected conserved orthologous regions. In this study, the most divergent *Fugu *orthologs were chosen as core sequences. The Gibbs sampling procedure searches for a common motif that has exactly one occurrence in the core sequence and no or one occurrence in the remainder of the sequences. After short motif seeds are identified, these are extended using a simple protocol to find larger conserved blocks: if the consensus score over a 5 nt region adjacent to the current motif exceeds a given threshold, the motif is extended with one nucleotide (in that direction). The larger a conserved block, the higher the confidence in the motif.

BlockSampler was run 100 times for each input set (subcluster plus *Fugu *ortholog) and corresponding random sets using default parameters; searching plus strand only (s = 0), prior set to 0.2, initial motif length of 8 nt. Only the threshold of the consensus score (default 1.0) was augmented to 1.2, selecting stronger conserved blocks. This generated 100 conserved blocks for each input set. To avoid redundancy, blocks overlapping more than 80% were merged. Concerning the benchmark datasets that consisted of only one distantly related ortholog, namely *Fugu*, we then selected those blocks that were conserved among all vertebrates under study. When studying more diverse datasets containing multiple distantly related species (with regard to mammals), we relaxed this requirement by allowing a block to be absent from one of the orthologs under study.

To account for the fact that short blocks are more likely to have a higher degree of conservation than long bocks, consensus scores [39] were compensated for their length. Blocks were then ranked according to this normalized consensus score (Cs_ad_), calculated using the formula Cs_ad _= (L/L+E)Cs, where L is the length of the conserved block, E is an empirical factor (set to 5) and Cs the consensus score.

To assess the relative individual contributions of the data reduction and motif detection steps to the final result, we applied BlockSampler on the full-length benchmark datasets. We used the same parameter setting as described above but, because of the longer sequence length in the full datasets, we increased the number of runs (1,000 runs for each benchmark dataset). Blocks were selected as described above. The best scoring 10% of the remaining blocks were searched for known motifs.

### Randomization

To set a threshold on the adapted consensus score of the blocks (blocks with a score above the threshold are considered relevant), we compared block scores of the genuine set with those of corresponding random sets. For each genuine dataset, 100 random sets were generated. A corresponding random set contains, besides the different homologous regions of the genuine subcluster under study, a random *Fugu *intergenic sequence. This additional random sequence was not orthologous with the mammalian sequences and thus is unlikely to contain the same motifs. In each random set, motifs were identified using the same procedure as described for the genuine set. For each random set the best scoring motif was selected, that is, the block with the highest normalized consensus score. This resulted in a group of the best scoring 100 false positive motifs. These scores were approximately normally distributed. As a threshold, we choose the 90th percentile of the best scoring random motifs.

### Motif validation

For each block we detected, a BLAT search against the human genome (May 2004 assembly) was performed [54,69]. This linked to the UCSC genome browser [51], where alignments between multiple vertebrate organisms were generated using MULTIZ [52]. Subsequently, we checked in the UCR browser [20] whether UCRs were identified in the intergenic regions under study.

To assess whether known transcription factor binding sites are located in the detected blocks, we compared the consensus sequence of each block with motifs described in the literature. In addition, we scanned the block consensus sequence with the Transfac 6.0 public database of vertebrate transcription factor binding site profiles [55]. This scanning was performed using MotifLocator [70-72] with a 0^th ^order vertebrate background model. Hits with a score >0.9 were regarded as potential binding sites. The binding sites are indicated by the Transfac factor name [55].

To calculate the statistical over-representation of homeodomain binding sites, 100 sequences were selected randomly from the *Fugu *genome and screened to make sure they differed from the genes under study. These random sequences were screened with matrix models from homeodomain binding sites (obtained from TRANSFAC 8.2) using MotifLocator, as described above. We calculated the chi-square statistic with Yates correction of the 2 × 2 contingency table test for the set of homeodomain binding sites [73]. Homeobox binding sites were significantly over-represented in a certain block at a *p* value of 10^-8^.

### Performance evaluation

To evaluate our newly developed procedure, we compared its performance to that of two algorithms often used for phylogenetic footprinting, namely the motif detection algorithm MotifSampler [39] and the multiple alignment procedures MAVID [10,65] and TBA [52]. These three algorithms were applied to the benchmark datasets and the resulting motifs (conserved in all organisms under study) were compared to those detected by the two-step procedure. We aligned the full-length initial datasets (Table 6) [44] using the online MAVID version at [74] with the default parameter setting [9].

Besides MAVID, we used TBA as it has been shown to outperform MAVID [52]. All the necessary tools were obtained from the Miller Lab website [75]. To generate a multiple alignment using TBA, we first pairwise aligned the initial datasets using blastz. We used the evolutionary tree ((human chimp)(rat mouse) *Fugu*); the additional blastz parameter file (latest version) was obtained from the E Margulies ftp site [76]. The final multiple alignment was obtained by running the TBA executable.

We applied MotifSampler both on the reduced datasets (subcluster + complete *Fugu *intergenic sequence [44]) and on the complete intergenic sequences (initial datasets). For the reduced sets we performed 100 MotifSampler runs, while for the complete datasets MotifSampler was run 1,000 times, each time using the standard parameter settings of the algorithm: the algorithm searches for only one motif (n = 1) of 8 nt (w = 8) on both strands (s = 1) and the prior probability of 1 motif copy (p) is 0.5. A third order vertebrate background model was used.

## Additional data files

The following additional data are available with the online version of this paper. Additional data file 1 contains the list of significant blocks detected in the six additional datasets and, for each block, the results of the Transfac screening. Additional data file 2 contains the stand-alone version of BlockSampler. Additional data file 3 contains the corresponding BlockSampler help file.

## Supplementary Material

Additional data file 1 The list of significant blocks detected in the six additional datasets and, for each block, the results of the Transfac screening Click here for file

Additional data file 2 The stand-alone version of BlockSampler Click here for file

Additional data file 3 The corresponding BlockSampler help file Click here for file

## Figures and Tables

**Table 1:** Conserved blocks detected in benchmark datasets

Gene	Number of blocks		
	
	Two-step	UCSC	UCR
*cfos*	0	0	0
*hoxb2*	8	5	0
*pax6*	13	11	0
*scl*	1	0	0
Total	22	16	0

Number of blocks two-step: number of conserved blocks identified using the two-step procedure. For more details on the blocks see Tables 2 (*hoxb2*), 3 (*pax6*) and 4 (*scl*). Number of blocks UCSC: the number of blocks detected by the two-step procedure that were recovered in the USCS genome browser (aligned between mammals and *Fugu*) [51]. Number of blocks UCR: the number of blocks detected by the two-step procedure that correspond to an ultra-conserved region [20].

**Table 2:** List of the significant blocks detected in the *hoxb2 *dataset

Block	Consensus sequence and possible binding sites
Hoxb2 1.1 (-)	**AATTCTTTGATGCAATCGGAGGGAGCTGTCAGGGGGCTAAGATTGATCGCCTCATsTCCT**
	*Meis (CTGTCA), CTGTCA: 26-31 +
	*Hox/Pbx, AGATTGATCG: 40-49 +
	**Cap**, M00253, NCANHNNN: 39-46 - (0.937); 22-29 - (0.918)
	**CDP CR1**, M00104, NATCGATCGS: 41-50 + (0.964)
	**CDP CR3+HD**, M00106, NATYGATSSS: 41-50 + (0.992)
	**CdxA**, M00101, AWTWMTR: 1-7 + (0.919); 6-12 + (0.903)
	**HSF2**, M00147, NGAANNWTCK: 40-49 + (0.925)
	**MEIS1**, M00419, NNNTGACAGNNN: 23-34 - (0.951)
	**TGIF**, M00418, AGCTGTCANNA: 24-34 + (0.966)
	**Pbx1**, M00096, ANCAATCAW: 39-47 - (0.909)
	
Hoxb2 2.1 (-)	**TTGCACTTrGAGTTTACATTTTAATG**
	*Octamer-motif (ATTTgCAT), GTTTACAT: 12-19 +
	*Adhf-2a (TGCACTgAGA), TGCACTTrGA: 2-11 +
	**CdxA**, M00101, AWTWMTR: 20-26 + (0.978); 19-25 - (0.905); 17-23 - (0.927)
	**SRY**, M00148, AAACWAM: 14-20 - (0.905)
	
Hoxb2 2.2 (UCSC)	**AAAAnTGTACTTTTTTAGTATTTACyT**
	*HoxA5 (TTTAaTAaTTA), TTTAGTATTTA: 14-24 +
	**CdxA**, M00101, AWTWMTR: 16-22 - (0.979)
	**SRY**, M00148, AAACWAM: 7-13 - (0.928)
	
Hoxb2 2.3 (UCSC)	**GTGTGTTCTAGTGAACATTTTCATATATATTTATTGGTTAT**
	*Glucocorticoid receptor, AGTGAACA: 10-17 +
	*CCAAT BOX, ATTGGTT: 27-33 +
	**Cap**, M00253, NCANHNNN: 15-22 + (0.919); 21-28 + (0.906); 7-14 - (0.919)
	**CdxA**, M00101, AWTWMTR: 23-29 + (0.958); 29-35 + (0.940); 28-34 - (0.956); 26-32 - (0.951); 24-30 - (0.958); 22-28 - (0.960)
	**FOXJ2**, M00422, NNNWAAAYAAAYANNNNN: 23-40 - (0.932)
	**HFH-3**, M00289, KNNTRTTTRTTTA: 25-37 + (0.908)
	**NF-Y**, M00185, TRRCCAATSRN: 30-40 - (0.914)
	**Oct-1**, M00162, CWNAWTKWSATRYN: 14-27 + (0.913)
	**Pbx-1**, M00096, ANCAATCAW: 30-38 - (0.948)
	
Hoxb2 2.4 (UCSC)	**GTGAACATTTTCATATATATTTATTGGTTATAGCCTGTTAAAATATTTTCTTTT**
	*GATA 1, TTATAGCC: 28-35 +
	*CCAAT BOX, ATTGGTT: 23-29 +
	**Cap**, M00253, NCANHNNN: 5-12 + (0.919); 11-18 + (0.906)
	**CCAAT box**, M00254, NNNRRCCAATSA: 21-32 - (0.940)
	**CdxA**, M00101, AWTWMTR: 13-19 + (0.958); 19-25 + (0.940); 39-45 + (0.925); 46-52 + (0.901); 36-42 - (0.930); 18-24 - (0.957); 16-22 - (0.951); 14-20 - (0.958); 12-18 - (0.960)
	**FOXD3**, M00130, NAWTGTTTRTTT: 41-52 + (0.924)
	**FOXJ2**, M00422, NNNWAAAYAAAYANNNNN: 13-30 - (0.932)
	**HFH-3**, M00289, KNNTRTTTRTTTA: 15-27 + (0.908)
	**HNF-3beta**, M00131, KGNANTRTTTRYTTW: 39-53 + (0.920)
	**NF-Y**, M00185, TRRCCAATSRN: 20-30 - (0.914)
	**Oct-1**, M00162, CWNAWTKWSATRYN: 4-17 + (0.913)
	**Pbx-1**, M00096, ANCAATCAW: 20-28 - (0.948)
	**SRY**, M00148, AAACWAM: 47-53 - (0.961)
	
Hoxb2 2.5 (UCSC)	**AATTCyCTCTTGGAACTTTCTTTGTTCTTCmGTAG**
	**HSF1**, M00146, AGAANRTTCN: 12-21 + (0.915); 12-21 - (0.930)
	**HSF2**, M00147, NGAANNWTCK: 12-21 + (0.948); 12-21 - (0.930)
	**SRY**, M00148, AAACWAM: 17-23 - (0.961)
	
Hoxb2 3.1 (UCSC)	**GGCCnAGACnAGCGATTGGCGGAGrCCGGTCCCGTGACCAnGAATTCCCTGyAATTT**
	NF-Y, M00185, TRRCCAATSRN: 12-22 - (0.915)
	**USF**, M00187, CYCACGTGNC: 29-38 - (0.957)
	**USF**, M00217, NCACGTGN: 30-37 + (0.902)
	
Hoxb2 3.2 (-)	**TCCCGTGACCAnGAATTCCCTGyAATTTCGnyGGAGTCC**
	USF, M00217, NCACGTGN: 1-8 + (0.902)

For each block, the consensus sequence is given followed by the possible binding sites situated in this block: motifs previously described in the literature [46] are marked with an asterisk. The motifs are summarized by their motif name (in bold), by their consensus sequence, if known, as described in the original article, by the sequence of the motif instance in our search, by the positions of the motif instance relative to the consensus sequence of the entire block and by the strand (indicated by a '+' or a '-') on which the motif occurred. Motif hits derived by Transfac are indicated by their matrix accession number, the consensus of this binding site and the instances of this motif in our search. These are further characterized by their positions relative to the consensus sequence of the entire block, by the strand on which the motif occurred and by the corresponding MotifLocator score (in parentheses). The blocks identified by the UCSC genome browser as conserved between mammals and *Fugu *are marked with 'UCSC', while the blocks detected by our two-step methodology but not present in the UCSC genome browser are indicated with a '-'.

**Table 3:** List of the significant blocks detected in the *pax6 *dataset

Block	Consensus sequence and possible binding sites
pax6 1.1 (UCSC)	CTTAATGATGAGAGATCTTTCCGCTCATTGCCCATTCAAATACAATTGTAGATCGAAGCCGGCCTT GTCAsGTTGAGAAAAAGTGAATTTCTAACATCCAGGACGTGCCTGTCTACT
	*Minimal fragment for expression in lens and cornea as described in [46]: 11-117 +
	**Cap**, M00253, NCANHNNN: 25-32 + (0.940); 79-86 - (0.964); 4-11 - (0.946); 1-8 - (0.903)
	**CCAAT box**, M00254, NNNRRCCAATSA: 27-38 + (0.901)
	*CdxA, M00100, 'MTTTATR': 1-7 + (0.921)*; 87-93 + (0.913)
	*CdxA, M00101, AWTWMTR: 1-7 + (0.934); 4-10 + (0.921); 38-44 + (0.905), 87-93 + (0.988)
	**c-Ets-1(p54)**, M00032, NCMGGAWGYN: 98-107 + (0.906)
	**c-Ets-1(p54)**, M00074, NNACMGGAWRTNN: 92-104 - (0.901)
	**En-1**, M00396, GTANTNN: 37-43 - (0.967)
	**GATA-3**, M00351, ANAGATMWWA: 11-20 + (0.920)
	**HSF2**, M00147, NGAANNWTCK: 13-22 - (0.933)
	**p53**, M00272, NGRCWTGYCY: 101-110 + (0.949)
	
pax6 1.2 (UCSC)	**CATTATTGTTGCCAGCACGAAGCATCACAATCAATCATAAGGAAGTCCAGTTGGCAGGTGTCAATCTTG**
	**CdxA**, M00101, AWTWMTR: 1-7 - (0.995)
	**Cap**, M00253, NCANHNNN: 25-32 + (0.934); 31-38 + (0.903); 35-42 + (0.903); 47-54 + (0.908); 61-68 + (0.937)
	**CDP CR3+HD**, M00106, NATYGATSSS: 27-36 - (0.907)
	**c-Ets-1(p54)**, M00074, NNACMGGAWRTNN: 36-48 + (0.902)
	*HOXA3, M00395, CNTANNNKN: 1-9 + (0.905)
	**MyoD**, M00184, NNCACCTGNY: 53-62 - (0.956)
	*Pbx-1, M00096, ANCAATCAW: 30-38 + (0.986); 2-10 - (0.923)
	**Sox-5**, M00042, NNAACAATNN: 3-12 - (0.932)
	**SRY**, M00148, AAACWAM: 33-39 + (0.910)
	**USF**, M00122, NNRNCACGTGNYNN: 51-64 + (0.913); 51-64 - (0.908)
	
pax6 1.3 (UCSC)	**GAAAAAGTGAATTTCTAACATCCAGGACGTGCCTGTCTACTTTCAGwGAATTGCATCCAATCACCCC**
	**Cap**, M00253, NCANHNNN: 3-10 - 0.964
	**CCAAT box**, M00254, NNNRRCCAATSA: 52-63 + (0.949)
	**CdxA**, M00100, 'MTTTATR': 11-17 + (0.913)
	**CdxA**, M00101, AWTWMTR: 11-17 + (0.988)
	**c-Ets-1(p54)**, M00032, NCMGGAWGYN: 22-31 + (0.906)
	**c-Ets-1(p54)**, M00074, NNACMGGAWRTNN:16-28 - (0.901)
	**En-1**, M00396, GTANTNN: 58-64 - (0.948)
	**GATA-1**, M00075, SNNGATNNNN: 56-65 - (0.930)
	**GATA-3**, M00077, NNGATARNG: 56-64 - (0.917)
	**NF-Y**, M00185, TRRCCAATSRN: 54-64 + (0.910)
	**p53**, M00272, NGRCWTGYCY: 25-34 + (0.949)
	**SRY**, M00148, AAACWAM: 59-65 + (0.917)
	
pax6 1.4 (UCSC)	**GTCTATATTTAATCCAATTATAAGGGTCACGGAGTAAGTGC**
	*Motif containing homeoboxes described in [46], TTTAATCCAATTATAA: 8-23 +
	**Cap**, M00253, NCANHNNN: 34-41 - (0.904)
	**CdxA**, M00100, 'MTTTATR': 16-22 + (0.907)
	**CdxA**, M00101, AWTWMTR: 16-22 + (0.995); 16-22 - (0.906); 6-12 - (0.931); 4-10 - (0.951)
	**En-1**, M00396, GTANTNN: 15-21 - (0.948)
	**Nkx2-5**, M00240, TYAAGTG: 34-40 + (0.927)
	**RORalpha1**, M00156, NWAWNNAGGTCAN: 18-30 + (0.919)
	**TCF11**, M00285, GTCATNNWNNNNN: 26-38 + (0.906)
	
pax6 1.5 (UCSC)	**GCATCCAATCACCCCCAGGG**
	**Cap**, M00253, NCANHNNN: 9-16 + (0.965)
	**En-1**, M00396, GTANTNN: 6-12 - (0.948)
	**GATA-3**, M00077, NNGATARNG: 4-12 - (0.917)
	**SRY**, M00148, AAACWAM: 7-13 + (0.917)
	
pax6 1.6 (UCSC)	**CAsGTTGAGAAAAAGTGAATTTCTAACATCCAGGACGTGCCTGTCTACTTTCAGw GAATTGCATCCAATCACCCCCAGGGAATTCnGCTAATGTCTCC**
	*Homeobox-binding site described in [46], GCTAATGTCTC: 87-97 +
	**Cap**, M00253, NCANHNNN: 69-76 + (0.965); 87-94 - (0.903); 11-18 - (0.964)
	**CCAAT box**, M00254, NNNRRCCAATSA: 60-71 + (0.949)
	**CdxA**, M00100, 'MTTTATR': 19-25 + (0.913)
	**CdxA**, M00101, AWTWMTR: 19-25 + (0.988)
	**c-Ets-1(p54)**, M00032, NCMGGAWGYN: 30-39 + (0.906)
	**c-Ets-1(p54)**, M00074, NNACMGGAWRTNN: 24-36 - (0.901)
	**En-1**, M00396, GTANTNN: 66-72 - (0.948)
	**GATA-1**, M00075, SNNGATNNNN: 64-73 - (0.930)
	**GATA-3**, M00077, NNGATARNG: 64-72 - (0.917)
	**NF-Y**, M00185, TRRCCAATSRN: 62-72 + (0.910)
	**p53**, M00272, NGRCWTGYCY: 33-42 + (0.949)
	**SRY**, M00148, AAACWAM: 67-73 + (0.917)
	
pax6 2.1 (UCSC)	**TGGGTCCATTTTCCAGAyGGTTTGTTACTCTTGCTGCmTGATTTrG**
	**Cap**, M00253, NCANHNNN: 6-13 + (0.921)
	**CdxA**, M00101, AWTWMTR: 9-15 + (0.918)
	**SRY**, M00148, AAACWAM: 21-27 - (0.942)
pax6 2.2 (-)	ATTTTGGTTGCTTTCAGGTwTAATTAACTTT
	**Nkx2-5**, M00241, CWTAATTG: 21-28 - (0.902)
	
pax6 2.3 (UCSC)	**ATTGTAATCATTTCAATTATCTTCA**
	**Cap**, M00253, NCANHNNN: 8-15 + (0.927)
	**En-1**, M00396, GTANTNN: 14-20 - (0.948)
	**Nkx2-5**, M00241, CWTAATTG: 14-21 - (0.930)
pax6 2.4 (-)	GGTTGCTTTCAGGTwTAATTAACTTTGAACAACAAATA
	**Nkx2-5**, M00241, CWTAATTG: 16-23 - (0.902)
	
pax6 3.1 (UCSC)	**TTGTAATTACTGCCCTTCATGTGGTCCGGTGCCTTGAACCATCTTTAATTAAAAGCATAATTAAGG**
	**AML-1a**, M00271, TGTGGT: 20-25 + (1.000)
	**Cap**, M00253, NCANHNNN: 39-46 + (0.910); 55-62 + (0.909); 6-13 - (0.916)
	**CdxA**, M00100, MTTTATR: 56-62 - (0.934)
	**CdxA**, M00101, AWTWMTR: 6-12 + (0.988); 44-50 + (0.913); 47-53 + (0.900); 48-54 + (0.905); 59-65 + (0.903); 60-66 + (0.926); 56-62 - (0.998); 47-53 - (0.913); 44-50 - (0.901); 43-49 - (0.907); 2-8 - (0.949);
	**En-1**, M00396, GTANTNN: 3-9 + (0.912); 4-10 - (0.912)
	**HSF2 **, M00147, NGAANNWTCK: 35-44 + (0.908)
	**Nkx2-5**, M00241, CWTAATTG: 56-63 + (0.935), 58-65 - (0.954)
	**USF**, M00217, NCACGTGN: 17-24 - (0.921)
	
pax6 3.2 (UCSC)	**AAGGCTTGCAGCTGCCTCCAAATCAATAGAyGTCAAAGAAATATGAAAACArTC**
	**CdxA**, M00101, AWTWMTR: 39-45 + (0.953); 36-42 - (0.925)
	**SRY**, M00148, AAACWAM: 35-41 + (0.961)
	**Cap**, M00253, NCANHNNN: 8-15 + (0.931); 39-46 - (0.940); 8-15 - (0.931)
	**AP-4**, M00175, VDCAGCTGNN: 7-16 - (0.902)
	**MyoD**, M00184, NNCACCTGNY: 7-16 + (0.957)
	**SRY**, M00160, NWWAACAAWANN: 19-30 + (0.928)
	
pax6 3.3 (UCSC)	**GCATAATTAAGGGAAGATCTAAAGAAAGACAATTACCAGATGGTCT**
	**Cap**, M00253, NCANHNNN: 1-8 + (0.909)
	**CdxA**, M00100, MTTTATR: 2-8 - (0.934)
	**CdxA**, M00101, AWTWMTR: 5-11 + (0.903); 6-12 + (0.926); 32-38 + (0.939); 2-8 - (0.998)
	**En-1**, M00396, GTANTNN: 30-36 - (1.000)
	**GATA-1**, M00075, SNNGATNNNN: 36-45 + (0.936)
	**GATA-2**, M00076, NNNGATRNNN: 36-45 + (0.922)
	**GATA-3**, M00351, ANAGATMWWA: 13-22 + (0.949)
	**HOXA3**, M00395, CNTANNNKN: 29-37 - (0.939)
	**Msx-1**, M00394, CNGTAWNTG: 30-38 - (0.915)
	**MyoD**, M00184, NNCACCTGNY: 35-44 - (0.919)
	**Nkx2-5**, M00241, CWTAATTG: 2-9 + (0.935); 4-11 - (0.954)
	**SRY**, M00148, AAACWAM: 21-27 + (0.961); 25-31 + (0.927)
	**USF**, M00122, NNRNCACGTGNYNN: 33-46 + (0.907); 33-46 - (0.904)

For each block, the consensus sequence is given followed by the possible binding sites situated in this block: motifs previously described in the literature [47] are marked with an asterisk. The motifs are summarized by their motif name (in bold), by their consensus sequence, if known, as described in the original article, by the sequence of the motif instance in our search, by the positions of the motif instance relative to the consensus sequence of the entire block and by the strand (indicated by a '+' or a '-') on which the motif occurred. Motif hits derived by Transfac are indicated by their matrix accession number, the consensus of this binding site and the instances of this motif in our search. These are further characterized by their positions relative to the consensus sequence of the entire block, by the strand on which the motif occurred and by the corresponding MotifLocator score (in parentheses). The blocks identified by the UCSC genome browser as conserved between mammals and *Fugu *are marked with 'UCSC', while the blocks detected by our two-step methodology but not present in the UCSC genome browser are indicated with a '-'.

**Table 4:** List of the significant blocks detected in the *scl *dataset

Block	Consensus sequence and possible binding sites
scl 1.1 (-)	**TTGCCAAATTAAAATGAATCATTTGGCCCATAATGGCCGAGGCGCT**
	*Conserved sequence identified in [47], GCCAAAT: 3-9 +
	*Putative SKN1 site reported in [47], AATGAATCATTT: 13-24 +
	**CdxA**, M00100, 'MTTTATR': 29-35 - (0.917)
	**CdxA**, M00101, AWTWMTR: 7-13 + (0.901); 8-14 + (0.905); 10-16 + (0.927); 29-35 + (0.927); 29-35 - (0.929); 7-13 - (0.913)
	*En-1, M00396, GTANTNN: 30-36 + (0.936)
	**Cap**, M00253, NCANHNNN: 19-26 + (0.932); 10-17 - (0.933)
	**Pbx-1**, M00096, ANCAATCAW:14-22 + (0.941)
	**AP-1**, M00199, NTGASTCAG: 14-22 + (0.913)
	*HOXA3, M00395, CNTANNNKN: 29-37 + (0.927)
	**Tst-1**, M00133, NNKGAATTAVAVTDN: 3-17 + (0.901)

For each block, the consensus sequence is given followed by the possible binding sites situated in this block: motifs previously described in the literature [48] are marked with an asterisk. The motifs are summarized by their motif name (in bold), by their consensus sequence, if known, as described in the original article, by the sequence of the motif instance in our search, by the positions of the motif instance relative to the consensus sequence of the entire block and by the strand (indicated by a '+' or a '-') on which the motif occurred. Motif hits derived by Transfac are indicated by their matrix accession number, the consensus of this binding site and the instances of this motif in our search. These are further characterized by their positions relative to the consensus sequence of the entire block, by the strand on which the motif occurred and by the corresponding MotifLocator score (in parentheses). The blocks identified by the UCSC genome browser as conserved between mammals and *Fugu *are marked with 'UCSC', while the blocks detected by our two-step methodology but not present in the UCSC genome browser are indicated with a '-'.

**Table 5:** Comparison of two-step procedure with other methodologies

Gene	Number of motifs	Two-step BS	BS	Two-step MS	MS	MAVID	TBA
*cfos*	2	0	0	0	0	0	0
*hoxb2*	17	8 (+5)	13	2	1	0	0
*pax6*	6	6	1*	0	0	6	6
*scl*	5	3 (+1)	1	0	0	0	0
Total	30	17 (+6)	15	2	1	6	6

Number of motifs: the number of motifs reported by Blanchette and Tompa [26] in *cfos*, Scemama *et al. *[46] in *hoxb2*, Kammandel *et al. *[47] in *pax6 *and Göttgens *et al. *[48] in *scl*. Two-step BS: the number of previously described motifs detected by the two-step procedure, combining data reduction and motif detection using BlockSampler. The numbers in parentheses are the number of motifs present in non-significant blocks. BS: the number of previously described motifs detected by BlockSampler in initial full-length datasets. Two-step MS: the number of previously described motifs detected by combining data reduction and motif detection using MotifSampler. MS: the number of previously described motifs detected by MotifSampler in initial full-length datasets. MAVID: the number of previously described motifs detected (correctly aligned) by MAVID. TBA: the number of previously described motifs detected by TBA. *Only part of a motif was detected.

**Table 6:** Base pair lengths of the intergenic sequences for each benchmark dataset

Gene	*Hs*	*Mm*	*Rn*	*Pt*	*Fr*
*cfos*	40,154	33,157	40,132	40,154	3,606*
					3,606^†^
					1,244^‡^
*hoxb2*	4,973	6,744	7,640	4,878	39,219
*pax6*	40,102	40,000	40,000	40,000	21,204
*scl*	20,981	16,471	20,343	39,999	20,155

The *Fugu cfos *intergenic sequences are derived from *SINFRUG00000132418, ^†^SINFRUG00000132419 and ^‡^SINFRUG00000143787. The Ensemble IDs (+ 1 Genebank accession number) are given in [56]. *Fr*,*Fugu rubripes*; *Hs*, *Homo sapiens*; *Mm*, *Mus musculus*; *Pt*, *Pan troglotydes*; *Rn*, *Rattus norvegicus*.
