# Supplementary material for: A novel approach to identifying regulatory motifs in distantly related genomes
Source: Genome Biol. 2005 Dec 30;6(13):R113. doi: 10.1186/gb-2005-6-13-r113 (PMC1414112; doi:10.1186/gb-2005-6-13-r113)
Supplement: Additional data file 1 — The list of significant blocks detected in the six additional datasets and, for each block, the results of the Transfac screening [file gb-2005-6-13-r113-S1.doc]

| EGR3 1.1 ^(a)^ | TGnCnCGCnGCCCynCGACCCTCCCnnCA ^(b)^ |
| --- | --- |
| EGR3 1.2 ^(a)^ | TTGTCTGTCCATATATGGnCAnCTACGTCAC ^(b)^ |
|  | **CdxA**, M00101, AWTWMTR: 11-17 + (0,960); 10-16 – (0,960) |
|  | **CREB, M00039, TGACGTMA: 23-30 – (0,954)** |

**Table A: List of the significant blocks detected in the *EGR3* data set.** For each block (a) the consensus sequence is given (b), followed by the possible binding sites situated in this block: Motif hits derived by Transfac are indicated by their matrix accession number, the consensus of this binding site and the instances of this motif in our search. These are further characterized by their positions relative to the consensus sequence of the entire block, by the strand on which the motif occurred and by the corresponding MotifLocator score. The strand is indicated by a “+” or a “-“.

| GSH1 1.1 ^(a)^ | TnTTnCGGCGTGGGTGGGGnTGACAAGAATAGAnTACATTATGCAGTTCATTTAGTTAACAAGTGAAATAATGnGGAAGCGTGCAGnGnGAATGCCnAGAGAA ^(b)^ |
| --- | --- |
|  | **Cap**, M00253, NCANHNNN: 43-50 + (0,919); 48-55 + (0,908); 59-66 – (0,954) |
|  | **CdxA,** M00100, MTTTATR: 37-43 + (0,917) |
|  | **CdxA**, M00101, AWTWMTR: 37-43 + (0,929); 67-73 + (0,995), 53-59 – (0,917); 37-43 – (0.927) |
|  | **EGR2,** M00246, NTGCGTRGGCGK: 6-17 + (0,912) |
|  | **EGR3**, M00245, NTGCGTGGGCGK: 6-17 + (0,917) |
|  | **Nkx2-5**, M00240, TYAAGTG: 59-65 + (0,934) |
|  | **SRY**, M00148, AAACWAM: 52-58 – (0,922); 46-52 – (0,912) |
| GSH1 1.2 ^(a)^ | TTAGTTAACAAGTGAAATAATGnGGAAGCGTGCAGnGnGAATGCCnAGAGAAAnGnnnnAAAnnCnnTnnnG ^(b)^ |
|  | **Cap**, M00253, NCANHNNN: 8-15 – (0,954) |
|  | **CdxA**, M00101, AWTWMTR: 16-22 + (0,995); 2-8 – (0,917) |
|  | **Nkx2-5**, M00240, TYAAGTG: 8-14 + (0,934) |
|  | **SRY**, M00148, AAACWAM: 1-7 – (0,922) |
| GSH1 1.3 ^(a)^ | AAAACCCTATTGAGAGnnnnnGGCCGCTnnnnGCGTAnn ^(b)^ |
| GSH1 1.4 ^(a)^ | AAAnTGAAAGAAAATGTTTTCCTATTACTTAATTCAATCAnAG ^(b)^ |
|  | **Cap**, M00253, NCANHNNN: 10-17 – (0,919) |
|  | **CdxA**, M00101, AWTWMTR: 24-30+ (0,923); 28-34 – (0,903); 23-29 – (0,921); 8-14 – (0,901) |
|  | **C/EBPalpha**, M00116, NNATTRCNNAANNN: 22-35 + (0,905) |
|  | **C/EBPbeta**, M00109, RNRTKDNGMAAKNN: 22-35 – (0,926) |
|  | **En-1**, M00396, GTANTNN: 22-28 – (0,927) |
|  | **NF-AT**, M00302, NANWGGAAAANN: 15-26 – (0,948) |
|  | **Nkx2-5**, M00240, TYAAGTG: 26-32 – (0,911) |
|  | **Nkx2-5**, M00241, CWTAATTG: 28-35 + (0,957) |
|  | **SRY**, M00148, AAACWAM: 7-13 + (0,961) |

**Table B: List of the significant blocks detected in the *GSH1* data set.** For each block (a) the consensus sequence is given (b), followed by the possible binding sites situated in this block: Motif hits derived by Transfac are indicated by their matrix accession number, the consensus of this binding site and the instances of this motif in our search. These are further characterized by their positions relative to the consensus sequence of the entire block, by the strand on which the motif occurred and by the corresponding MotifLocator score. The strand is indicated by a “+” or a “-“.

| HOXB5 1.1 ^(a)^ | GTCATnATTTGTAACCATAGAGCATGAATTACCTCTTGAnGTCATCAGnGAGAATTTACGACTGGTCAACAAAnGCACGTGAT ^(b)^ |
| --- | --- |
|  | **Cap**, M00253, NCANHNNN: 58-65 – (0,949) |
|  | **CdxA,** M00100, MTTTATR: 7-13 + (0,929); 54-60 + (0,941) |
|  | **CdxA**, M00101, AWTWMTR: 54-60 + (0,958) |
|  | **En-1**, M00396, GTANTNN: 26-32 – (0,950) |
|  | **HSF2** , M00147, NGAANNWTCK: 51-60 + (0,919) |
|  | **USF**, M00217, NCACGTGN: 75-82 + (0,929); 75-82 – (0,982) |
| HOXB5 1.2 ^(a)^ | CnCCCATATTTGGCCGCATACATAGCAAA ^(b)^ |
|  | **Cap**, M00253, NCANHNNN: 16-23 + (0,901) |
|  | **CdxA**, M00101, AWTWMTR: 5-11 – (0,953) |
|  | **En-1**, M00396, GTANTNN: 6-12 + (0,915) |
|  | **HOXA3**, M00395, CNTANNNKN: 5-13 + (0,948) |
| HOXB5 1.3 ^(a)^ | TAATTCATTAATACATCATAAATCGTGAAGCACAGGGTTATAACGACCAnGATCnACAAATCAAGCCCTCnAAAA ^(b)^ |
|  | **Cap**, M00253, NCANHNNN: 5-12 + (0,924); 16-23 + (0,901) |
|  | **CdxA,** M00100, MTTTATR: 7-13 + (0,911); 7-13 – (1,00); 6-12 – (0,938) |
|  | **CdxA**, M00101, AWTWMTR: 7-13 + (1,00); 39-45 + (0,925); 17-23 – (1,00); 6-12 – (1,00) |
|  | **Pbx-1**, M00096, ANCAATCAW: 56-64 + (0,912) |
|  | **SRY**, M00148, AAACWAM: 59-65 + (0,901) |
| HOXB5 1.4 ^(a)^ | AAACGAAGTACAGTGCATnGCTATAATTCATTAATACATCATAAATCGTGAAG ^(b)^ |
|  | **Cap**, M00253, NCANHNNN: 28-35 + (0.924); 39-46 + (0,901) |
|  | **CdxA,** M00100, MTTTATR: 30-36 + (0,911); 40-46 – (1,00); 29-35 – (0,938); 22-28 – (0,907) |
|  | **CdxA**, M00101, AWTWMTR: 22-28 + (0,906); 30-36 + (1,00); 40-46 – (1,00); 29-35 – (1,00); 22-28 – (0,995) |
|  | **SRY**, M00148, AAACWAM: 1-7 + (0,907) |
| HOXB5 1.5 ^(a)^ | AAACGAAGTACAGTGCATnGCTATAATTCATTAATACATCATA ^(b)^ |
|  | **Cap**, M00253, NCANHNNN: 28-35 + (0,924) |
|  | **CdxA,** M00100, MTTTATR: 30-36 + (0,911); 29-35 – (0,938); 22-28 – (0,907); |
|  | **CdxA**, M00101, AWTWMTR: 22-28 + (0,906);30-36 + (1,00); 29-35 – (1,00); 22-28 – (0,995) |
|  | **SRY**, M00148, AAACWAM: 1-7 + (0,907) |
| HOXB5 1.6 ^(a)^ | ACGAAGTACAGTGCATnGCTATAATTCATTAATACATCATAAATCGTGA ^(b)^ |
|  | **Cap**, M00253, NCANHNNN: 26-33 + (0,924); 37-44 + (0,900) |
|  | **CdxA,** M00100, MTTTATR: 28-34 + (0,911); 38-44 – (1,00); 27-33 – (0,938); 20-26 – (0,907) |
|  | **CdxA**, M00101, AWTWMTR: 20-26 + (0,906); 28-34 + (1,00); 38-44 – (1,00); 27-33 – (1,00);  20-26 – (0,995) |

**Table C: List of the significant blocks detected in the *HOXB5* data set.** For each block (a) the consensus sequence is given (b), followed by the possible binding sites situated in this block: Motif hits derived by Transfac are indicated by their matrix accession number, the consensus of this binding site and the instances of this motif in our search. These are further characterized by their positions relative to the consensus sequence of the entire block, by the strand on which the motif occurred and by the corresponding MotifLocator score. The strand is indicated by a “+” or a “-“.

| MEIS2 1.1 ^(a)^ | TAACCCCAAAATGACCCCAATTTGACACCrCAAGATGAAATTTTACCGCCTGTTAAAACCA ^(b)^ |
| --- | --- |
|  | **Cap**, M00253, NCANHNNN: 31-38 – (0,930); 7-14 – (0,942) |
|  | **CdxA**, M00101, AWTWMTR: 42-48 + (0,917); 51-57 – (0,930) |
|  | **RORalpha1**, M00156, NWAWNNAGGTCAN: 11-23 – (0,911) |
|  | **TCF11**, M00285, GTCATNNWNNNNN: 3-15 – (0,980) |
| MEIS2 1.2 ^(a)^ | ATGAAATTTTACCGCCTGTTAAAACCATTTCCAGCCTGGGCnn ^(b)^ |
|  | **Cap**, M00253, NCANHNNN: 25-32 + (0,915); 31-38 + (0,926); |
|  | **CdxA**, M00101, AWTWMTR: 8-14 + (0,917); 27-33 + (0,942); 17-23 – (0.930) |
|  | **En-1**, M00396, GTANTNN: 25-31 – (0,911) |

**Table D: List of the significant blocks detected in the *MEIS2* data set.** For each block (a) the consensus sequence is given (b), followed by the possible binding sites situated in this block: Motif hits derived by Transfac are indicated by their matrix accession number, the consensus of this binding site and the instances of this motif in our search. These are further characterized by their positions relative to the consensus sequence of the entire block, by the strand on which the motif occurred and by the corresponding MotifLocator score. The strand is indicated by a “+” or a “-“.

| PCDH8 1.1 ^(a)^ | CTAACGAGGGCTTCATAAGCCTTTGATACAGTCTGATCTTTGAAAC ^(b)^ |
| --- | --- |
|  | **Cap**, M00253, NCANHNNN: 13-20 + (0,923); 28-35 + (0,936); 29-36 – (0,956) |
|  | **CdxA**, M00101, AWTWMTR: 22-28 + (0,901) |
|  | **GATA-2**, M00349, ASAGATAANA: 32-41 – (0,935) |
|  | **GATA-3**, M00350, NGAGATAANA: : 32-41 – (0,918) |
|  | **GATA-3**, M00351, ANAGATMWWA: 32-41 – (0,965) |
|  | **SRY**, M00148, AAACWAM: 22-28 – (0,906) |

**Table E: List of the significant blocks detected in the *PCDH8* data set.** For each block (a) the consensus sequence is given (b), followed by the possible binding sites situated in this block: Motif hits derived by Transfac are indicated by their matrix accession number, the consensus of this binding site and the instances of this motif in our search. These are further characterized by their positions relative to the consensus sequence of the entire block, by the strand on which the motif occurred and by the corresponding MotifLocator score. The strand is indicated by a “+” or a “-“.
