# Supplementary material for: A novel approach to identifying regulatory motifs in distantly related genomes
Source: Genome Biol. 2005 Dec 30;6(13):R113. doi: 10.1186/gb-2005-6-13-r113 (PMC1414112; doi:10.1186/gb-2005-6-13-r113)
Supplement: Additional data file 3 — The corresponding BlockSampler help file [file gb-2005-6-13-r113-S3.doc]

# Overview

**BlockSampler** is used to find conserved blocks in the upstream region of sets of orthologous genes.

Some basic remarks on the program:

- The program should be started from the command line. A full description of the required and optional arguments can be found below.
- The final results are printed either on STDOUT or in a file in GFF format.
- The block models found can be saved in a separate file using the **-m** switch. To further analyze these matrices you can download MotifRanking from the INCLUSive website [1,2,3].
- On the STDERR you can monitor the progress of the program.

# Required arguments

###

| **Switch** | **Argument** | Description |
| --- | --- | --- |
| **-f** | file | Input sequences in fasta format. There should be at least 2 sequences in the file. |
| **-b** | file | File containing a list of sequence ids and the names referring to the related background model files. Format description of this file can be found below. |
| **-i** | value | Defines the root sequence of the data set. This value should be similar to one of the identifiers of the sequences in the fasta file. |

# Optional arguments

| **Switch** | **Argument** | **Description** |
| --- | --- | --- |
| **-s** | 0|1 | Select strand. (default plus strand). 0 is only input sequences, 1 include reverse complement. |
| **-p** | value | Sets prior probability of finding one instance of the block. This value allows the user to define the required characteristics of the block to search for. If the prior is set close to 0 then more conserved blocks are retrieved, increasing the prior will introduce more degeneracy into the block model. If the prior is set too small, it is possible that no block is found. Default = 0.2. |
| **-w** | value | Sets length of the initial seed of the block (default 8). |
| **-r** | value | Set number of times the BlockSampler should be repeated (default = 100). When using this option it is best to define a matrix file with the '-m' switch to store the block models generated at each repetition. This file can later be used to analyze the block models and select the best scoring models |
| **-t** | value | Sets threshold to extend block length (a value between 0 and 2, default = 1.0). |

# Output definitions

| **Switch** | **Argument** | **Description** |
| --- | --- | --- |
| **-o** | file | Sets the output file to save the results. The found block instances are written to this file in GFF format. Default the results are written to STDOUT |
| **-m** | file | Sets the file name of the matrix file to store the retrieved block models. If not provided the matrices are not saved.  This matrix file can be used with the MotifScanner [3] to screen DNA sequences for instances of the retrieved blocks.  If you have done multiple runs (switch '-r'), you should use this matrix file to further analyze the results. |

#

# Background model descriptions

In BlockSampler each orthologous intergenic sequence in the input data set is scored with its appropriate species-specific background model (structure is given below). In order to provide information about which sequence should be scored with which background model, a file containing links to the different background models is required (see parameter –b).

ENSG00000173917 /path/to/your/backgrounddir/homo_sapiens_order.bg

ENSPTRG00000009352 /path/to/your/backgrounddir/pan_troglodytes_order.bg

ENSMUSG00000047830 /path/to/your/backgrounddir/mus_musculus_order.bg

ENSRNOG00000008365 /path/to/your/backgrounddir/rattus_norvegicus_order.bg

SINFRUG00000136637 /path/to/your/backgrounddir/fugu_rubripes_order.bg

A background model is stored as an ascii text file using a well defined format. Below you can find an example of the first-order *Homo sapiens* background model file. The file should always start with the word #INCLUSive at the first position of the file. Next, there are several lines describing the organism, data set and order of the background model. Finally the data itself is represented.

--

#INCLUSive Background Model v1.0

#

#Order = 1

#Organism = human

#Sequences = d:\sae\projects\sista.sequence\sequenceviewer\bgModels\epd_homo_sapiens_499_chromgenes_non_split.tfa

#Path =

#

#snf

0.2570 0.2534 0.2465 0.2432

#oligo frequency

0.2570

0.2534

0.2465

0.2432

#transition matrix

0.3121 0.1944 0.2751 0.2184

0.2751 0.3014 0.1547 0.2688

0.2400 0.2718 0.2943 0.1939

0.1970 0.2469 0.2637 0.2924

--

You can get some pre-compiled background models at our Background Model download page [3].

To create your own background model you can use the program CreateBackgroundModel which you can find on the INCLUSive website [3].

# Example

Here is a step-by-step example on how to use the BlockSampler. The current version is a Linux version. To make sure that all the file specifications are clear, an example data set is provided on the supplementary website [4], together with the background model files.

## 1. Software installation

The first step is the installation of the program. Download our software provided as additional data file or at the supplementary website [4].

If you download the stand-alone from the Genome Biology website (as additional data file) make sure that you rename it from 'gb-2005-6-13-r113-S2.af2' to ‘BlockSampler’ (no file extension).

mv gb-2005-6-13-r113-S2.af2 BlockSampler

If you save it, make it executable (chmod 755 BlockSampler) and make sure that the program is included in your path. You can test if it works by just typing BlockSampler at the prompt without any option. The output should look like this:

ssh|rvanhell>BlockSampler

Seed = 750317702

Usage: BlockSampler <ARGS>

Required Arguments

-f <fastaFile> Sequences in FASTA format

-b <bgFile> File containing a list of sequence ids and background model file names.

-i <value> Defines the root sequence of the data set. <value> should be similar to the identifier of the sequence in the fasta file.

Optional Arguments

-s <0|1> Select strand. (default plus strand)

0 is only input sequences, 1 include reverse complement.

-p <value> Sets prior probability of 1 motif copy. (default 0.2).

-w <value> Sets length of the motif (default 8).

-r <runs> Set number of times the MotifSampler should be repeated

(default = 1).

-t <value> Sets threshold to extend motif length (default = 1.0).

Output formatting Arguments

-o <outFile> Output file to write results (default stdout).

-m <matrixFile> Output file to write retrieved motif models.

Version 3.1 -- the bug fix release

Questions and Remarks: [gert.thijs@esat.kuleuven.ac.be](mailto:gert.thijs@esat.kuleuven.ac.be)

## 2. Input Sequences

Input sequence should be in fasta format. An example is provided on the supplementary website [4].

## 3. Background Model

For this example we use the third-order background model from different vertebrate organisms: The sequence with identifier ENSG00000007372 is derived from *Homo sapiens* and is thus scored by a *Homo sapiens*-specific background model, namely homo_sapiens_3.bg. In a similar way, each ortholog is scored with its species-specific background model. How to download or create background models is explained above. An example of a background file can be downloaded from the supplementary website.

## 4. Do a single run

First, we do a simple test in which we test one set of parameters in one single run. We use default parameters except for

- **-t 1.2** Here we augment the threshold of the consensus score. This will allow the algorithm to find stronger conserved blocks.
- -**r 1** we only perform one BlockSampler run.

**Command line**: BlockSampler -f example.fasta -b example.bg -i SINFRUG00000121553 -t 1.2 -r 1 >error1.log

Note that in this example the output is written on STDOUT and the STDERR is redirected to 'error1.log'

#INCLUSive GFF File

#id: block_SINFRUG00000121553_1 consensus: CATTATTGTTGCCAGCACGAAGCATCACAATCAATCATAAG sequences: 5 instances: 5 cs: 1.51 ic: 1.50 ll: 264.17

ENSRNOG00000004410 BlockSampler misc_feature 320 360 2.08702e+23 + . id "block_SINFRUG00000121553_1"; site "CATTATTGTTGCCAGCACGAAGCATCACAATCAATCATAAG";

ENSMUSG00000027168 BlockSampler misc_feature 315 355 3.07531e+23 + . id "block_SINFRUG00000121553_1"; site "CATTATTGTTGCCAGCACGAAGCATCACAATCAATCATAAG";

ENSPTRG00000003474 BlockSampler misc_feature 950 990 1.7212e+23 + . id "block_SINFRUG00000121553_1"; site "CATTATTGTTGCCAGCACGAAGCATCACAATCAATCATAAG";

ENSG00000007372 BlockSampler misc_feature 949 989 2.94992e+23 + . id "block_SINFRUG00000121553_1"; site "CATTATTGTTGCCAGCACGAAGCATCACAATCAATCATAAG";

SINFRUG00000121553 BlockSampler misc_feature 10098 10138 1.64152e+21 + . id "block_SINFRUG00000121553_1"; site "CATTATTGTTGCCAACACGAAGCATCAGAATCAATCACGAG";

## 5. Do a batch run and store motif models

Once we have tested a few parameter settings in single runs, it is time to move on and do some more extensive tests. Here we will repeat the same experiment 50 times and save the found matrices to a separate file. You can try the following parameter settings:

- **-p 0.3**  the prior probability of finding one copy of the block is set to 0.3.
- **-t 1.5** Here we augment the threshold of the consensus score even further. This will allow the algorithm to find stronger conserved blocks.
- **-r 50**  the test is repeated 50 times
- **-o example50.gff**  output is written in gff file
- **-m example50.mtrx**  block models are written to a matrix file

**Command line**: BlockSampler -f example.fasta -b example.bg -i SINFRUG00000121553 -t 1.5 -r 50 -p 0.3 -o example50.gff -m example50.mtrx >error50.log

Note that in this example the output is written to 2 files, one gff and one matrix. The STDERR is redirected to 'error50.log'.

The output files 'example50.gff' and 'example50.mtrx' can be downloaded from the supplementary website [4].

### References
